# MOB kinase activator 1A acts as an oncogene by targeting PI3K/AKT/mTOR in ovarian cancer

**DOI:** 10.1007/s12672-023-00705-3

**Published:** 2023-06-14

**Authors:** Jian Lei, Jing-Ying Xu, Min Hu, San-Gang Wu, Juan Zhou

**Affiliations:** 1 Department of Obstetrics and Gynecology, the First Affiliated Hospital of Xiamen University, School of Medicine, Xiamen University, Xiamen, 361003 China; 2 Department of Obstetrics and Gynecology, Fujian Medical University Union Hospital, Fuzhou, 350001 China; 3 Department of Radiation Oncology, the First Affiliated Hospital of Xiamen University, School of Medicine, Xiamen University, Xiamen, 361003 China

**Keywords:** MOB1A, Ovarian cancer, Proliferation, Autophagy

## Abstract

**Background:**

To illuminate the precise roles of MOB Kinase Activator 1 A (MOB1A) in the development of ovarian cancer (OC).

**Methods:**

MOB1A expression and clinical data of OC were obtained from the public database on gene expression and proteomics. Meanwhile, verification of expression was carried out in Gene Expression Omnibus, the Human Protein Atlas, and OC cell lines. The prognosis of MOB1A was explored in the Kaplan-Meier plotter. RNA interference and lentivirus vectors were applied to construct knockdown and overexpressed cell models. Changes in the malignant behaviors of OC cells were detected by cholecystokinin octopeptide cell counting kit, wound healing, colony formation assay, transwell, flow cytometry assays, and in vivo experiments. Changes in proteins in the PI3K and autophagy-related makers were detected by western blot analysis.

**Results:**

The expression of MOB1A was significantly upregulated and accompanied by an inferior survival rate in OC. Knockdown of MOB1A inhibited the proliferation, invasion, migration, and cell cycle of OC cells, whereas induced cell autophagy. MOB1A upregulation had the opposite effects. In addition, bioinformatics analysis and western blot experiments showed that MOB1A plays an important role in the PI3K/AKT/mTOR pathway.

**Conclusions:**

Our findings indicated that MOB1A is highly expressed and related to poor prognosis in OC. MOB1A plays a role in promoting the malignant biological behavior of tumor cells through PI3K/AKT/mTOR signaling pathway.

**Supplementary Information:**

The online version contains supplementary material available at 10.1007/s12672-023-00705-3.

## Introduction

Ovarian cancer (OC) is one of the most lethal gynecologic malignancies in women [1]. Due to a lack of specific symptoms and effective screening methods, approximately three-quarters of OC patients were diagnosed with advanced-stage disease (stage III-IV cancer). Surgery and platinum/taxane-based chemotherapy remain the main treatment strategy for advanced-stage OC [2]. However, three-quarters of patients still die of disease recurrence after receiving multimodal therapy [3]. Therefore, potential biomarkers regarding prognostication and therapeutic targets are important for the management of OC.

MOB kinase activator 1A (MOB1A) is one of the members of the family of Mps One binder (MOB) coactivator proteins, which was first explored in 2004 [4]. MOB1A is a mainly cytoplasmic protein and is known to control organ size and tumor growth [5]. Prior study has shown that MOB1A was involved in the Hippo signaling pathway and could hyperactivate NDR/LATS kinases [6]. However, the exact mechanism remains to be elucidated. Dysregulation of MOB1A has been reported in several malignant tumors, including colorectal cancer, glioblastoma, intrahepatic cholangiocarcinoma, non-small cell lung cancer, and gallbladder carcinoma [7–11]. However, the role of MOB1A in OC remains unclear. In addition, the exact mechanism of MOB1A in cancer development remains to be elucidated.

In this study, using the public datasets, we found that MOB1A was significantly upregulated in OC tissues compared with normal ovarian tissues. The purpose of the present study was to elucidate the effect of MOB1A on the development of OC. We found that overexpression of MOB1A was associated with inferior survival in OC. In addition, inhibition of MOB1A inhibited the malignant process of OC cells and induced cell autophagy. Moreover, we also found that MOB1A plays an important role in PI3K/AKT/mTOR signaling pathway.

## Results

### Identification of overexpressed genes in OC using public datasets

**Fig. 1:**
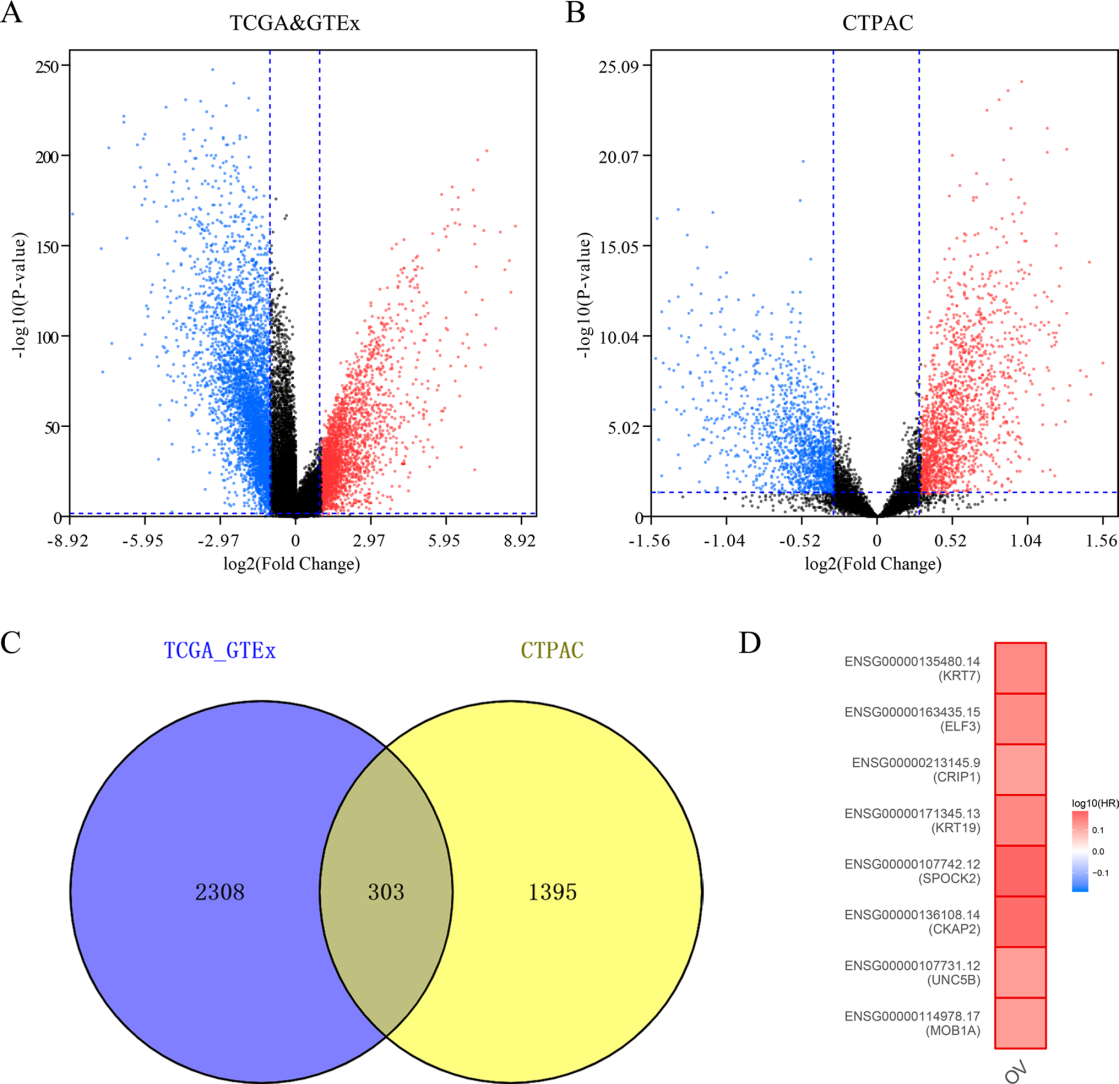
Differential expression genes in ovarian cancer (TCGA, GTEx, and CTPAC). **A**-**B** The volcano plots visualize the differential expression genes in TCGA and CTPAC, respectively; **C** Venn diagram for the intersection of upregulated genes; **D** Prognostic values of the eight key genes in ovarian cancer

Using the The Cancer Genome Atlas (TCGA), the Genotype-Tissue Expression (GTEx), and Clinical Proteomic Tumor Analysis Consortium (CPTAC) data sets, a total of 464 OC samples and 110 normal ovarian samples were explored. There were 2611 high-expression genes screened from the TCGA and GTEx data sets. Moreover, 1698 upregulated genes in the protein level were separated from the CTPAC data set. The volcano plots of differentially expressed genes (DEGs) among each data set are shown in Fig. 1A and B. By Venn diagram analysis, 303 common genes in the intersection of the three data sets were identified and selected for further analysis (Fig. 1C).

### MOB1A affects the survival of OC

**Fig. 2:**
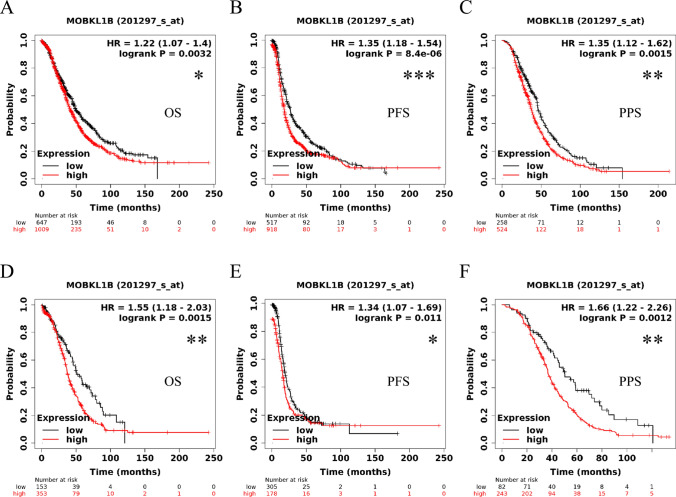
Prognostic values of MOB1A in ovarian cancer. **A**–**C** Prognostic values (OS, PFS, PPS) of MOB1A in ovarian cancer from KM; **D**–**F** Prognostic values (OS, PFS, PPS) of MOB1A in TP53-mutated ovarian cancer from KM. *P < 0.05, **P < 0.01, ***P < 0.001

To identify the key genes more accurately in OC, we explored the prognosis of the higher expression genes by using Gene Expression Profiling Interactive Analysis (GEPIA). Eight genes (KRT7, ELF3, CRIP1, KRT19, SPOCK2, CKAP2, UNC5B, and MOB1A) associated with poor prognosis were identified (Fig. 1D). MOB1A, also known as MOBKL1B, was selected for further survival analysis using The Kaplan-Meier (KM) plotter. The results showed that the overexpression of MOB1A was associated with shorter overall survival (OS), progress-free survival (PFS), and post-progression survival (PPS) in OC patients and TP53 mutations in OC patients (Fig. 2). The multivariable Cox proportional hazard model included clinical factors, degree of immune cell infiltration as well as MOB1A expression also validated MOB1A as an independent prognostic factor for OC patients (Table 1).

**Table 1:** A multivariable Cox proportional hazard model of MOB1A in OC patients from TCGA

Variables	HR (95% CI)	*P* value
Age	1.03 (1.01–1.04)	< 0.001
race (Black)	1.32 (0.51–3.40)	0.566
race (White)	0.85 (0.38–1.94)	0.705
Purity	0.13 (0.04–0.46)	0.001
B_cell	0.83 (0.00-627.26)	0.957
CD8_Tcell	0.33 (0.01–14.66)	0.564
CD4_Tcell	0.00 (0.00-0.02)	0.002
Dendritic	0.31 (0.01–15.76)	0.561
MOB1A	1.44 (1.16–1.79)	0.001

### MOB1A is highly expressed in OC

**Fig. 3:**
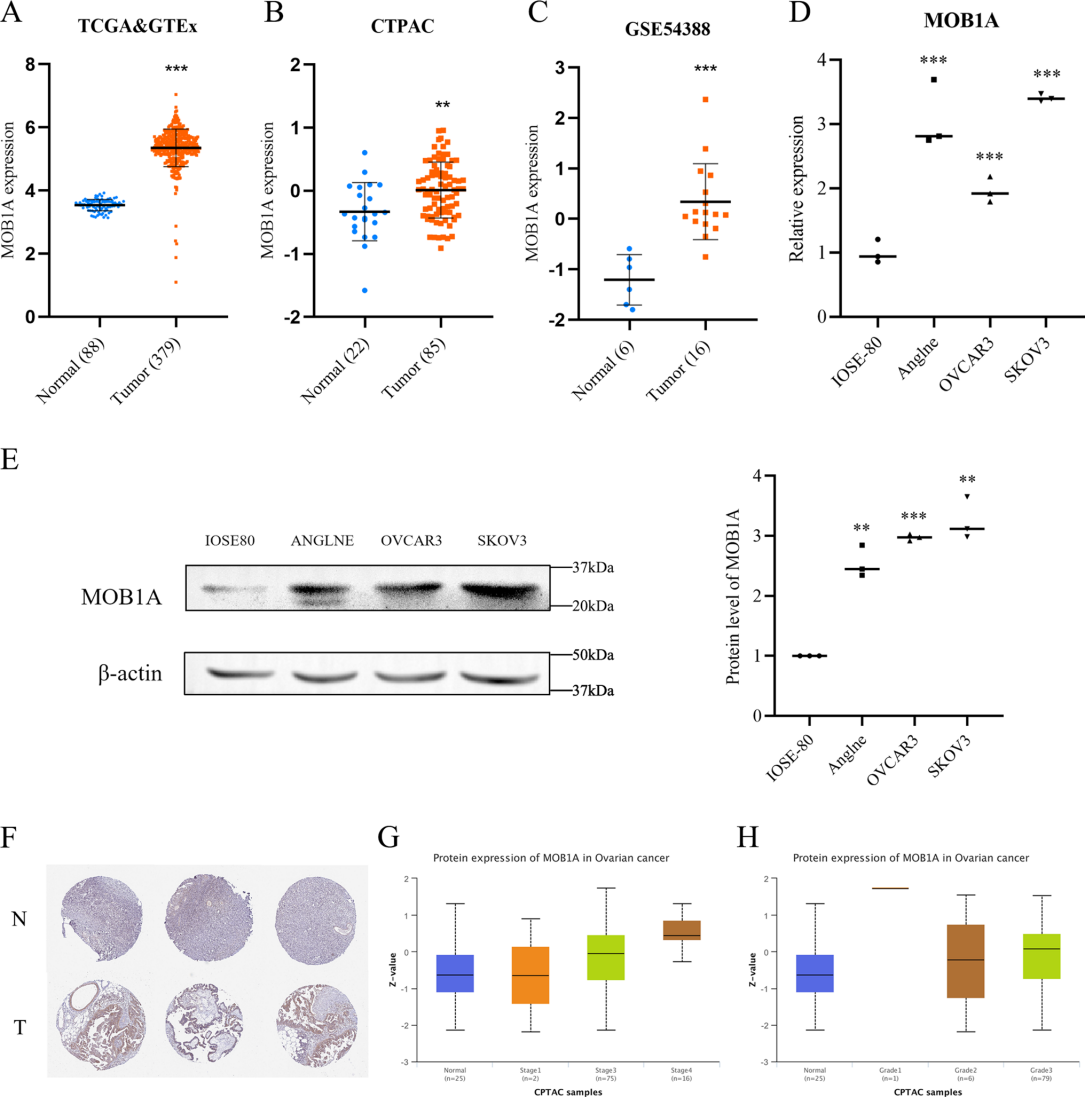
Expression of MOB1A in ovarian cancer. **A**–**C** The expression of MOB1A in ovarian normal and tumor tissues (mean ± SD); **D**, **E** The mRNA and protein expression of MOB1A in ovarian cancer cells (mean, N = 3); **F** Representative immunohistochemistry images of MOB1A protein expression in ovarian cancer from HPA, N = normal, T = tumor; **G**, **H** Protein expression of MOB1A in different stages and grades of ovarian cancer tissues (median with interquartile and range). **P < 0.01, ***P < 0.001

Further, we combined data from GSE54388 as external validation to illustrate the role of high expression of MOB1A in OC (Fig. 3A C). To explore the mechanism of action of MOB1A on the biological function of OC cells, the expression levels of MOB1A were evaluated in three different OC cell lines (Anglne, OVCAR3, and SKOV3) and one normal ovarian line (IOSE80) using real-time polymerase chain reaction (PCR) and western blot (WB). The OC cell lines, especially the SKOV3 cells, showed a significantly higher level of MOB1A expression than the normal ovarian cell line (Fig. 3D and E). Therefore, SKOV3 cells were selected for the subsequent experiments. The matching high expression occurred in OC patients with immunohistochemical results from the Human Protein Atlas (HPA) (Fig. 3F). The expression of MOB1A was positively correlated with the tumor stage and histological grade of OC patients (Fig. 3G H).

### Downregulation of MOB1A suppresses ovarian carcinoma cell proliferation, migration, invasion, and cell cycle

**Fig. 4:**
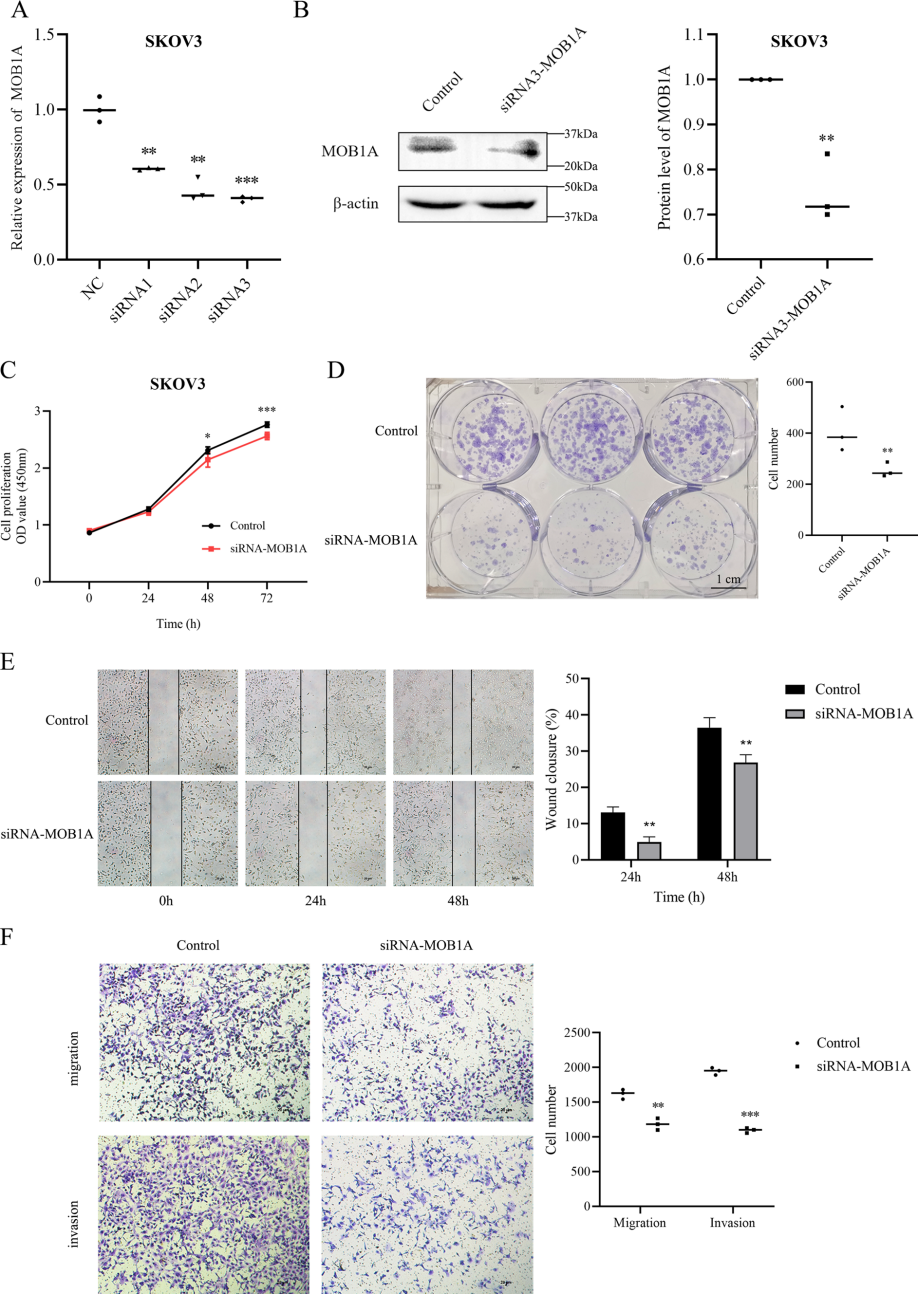
MOB1A downregulation suppressed ovarian carcinoma cell proliferation, migration, invasion ability, and cell cycle. Changes in the malignant behaviors of ovarian cancer cells were detected by CCK8, wound healing, colony formation assay, transwell, and flow cytometry assays, respectively. Results showed the inhibition of MOB1A with siRNA transfection (**A**, **B**) reduced the proliferation (**C**, **D**), migration, and invasion (**E**, **F**) of SKOV3 cells compared with the control groups (mean, N = 3). Bar scale: 1 cm (D), 20 μm (**E** and **F**). **P < 0.01, ***P < 0.001

Analysis by qRT-PCR and WB indicated that transfection of small interfering RNA (siRNA) targeting MOB1A reduced the expression of MOB1A. Our RT-PCR assays indicated that the viability of si-MOB1A-transfected cells was continuously lower than mock-transfected cells (Fig. 4A and B). The siRNA3 with a suitable knockdown effect was used for further experiments. Cholecystokinin octopeptide cell counting kit (CCK-8) staining demonstrated significantly reduced cell proliferation capacity in siRNA-MOB1A-transfected cells (Fig. 4C). We also detected the colony–forming ability of SKOV3 cells by plate clone formation assay. The results of plate cloning showed that the colony formation in the si-MOB1A group was decreased (Fig. 4D).

Wound-healing assay showed that MOB1A downregulation reduced cell migration compared with the control and mock-transfected cells (Fig. 4E). Transwell assays showed that cells transfected with si-MOB1A had reduced migration and invasion ability compared with control and mock-transfected cells (Fig. 4F). These findings indicated that MOB1A knockdown was able to depress cell migration and invasion in SKOV3 cells.

**Fig. 5:**
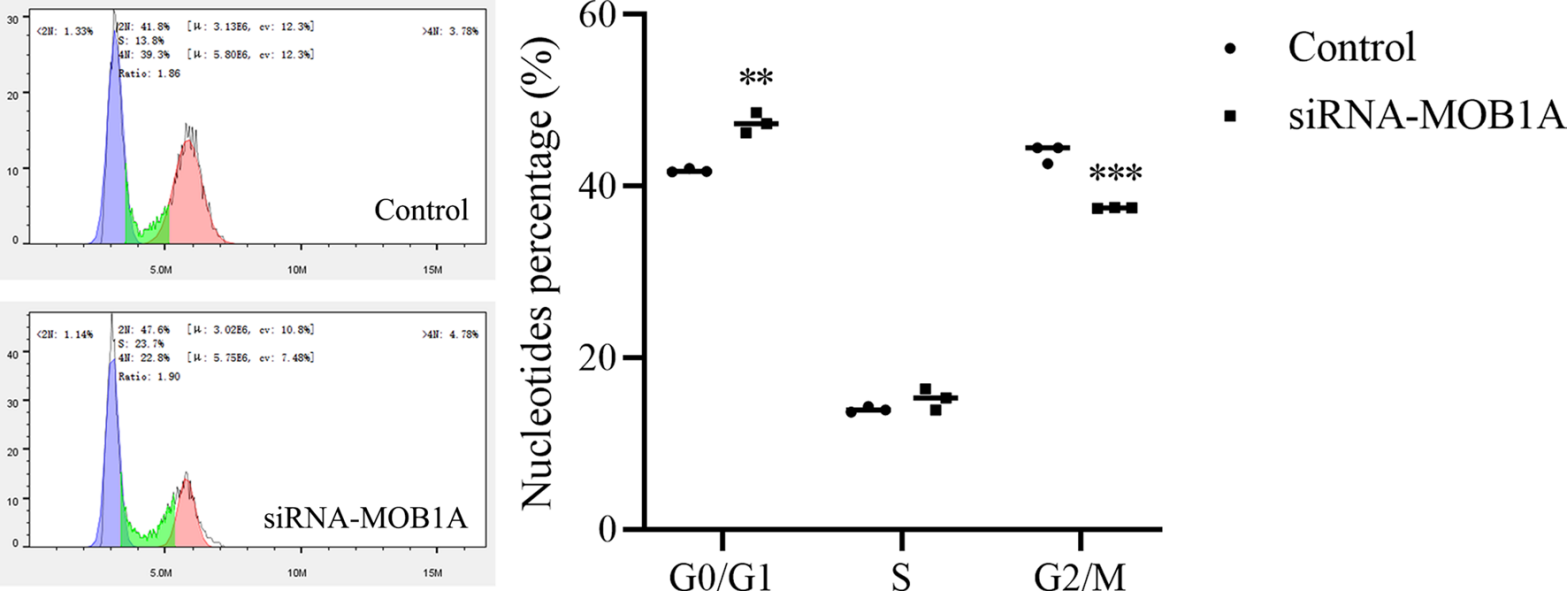
Knockdown of MOB1A inhibited cell cycle progression in ovarian cancer (mean, N = 3). **P < 0.01, ***P < 0.001

Cell cycle assays showed that MOB1A downregulation induced G1/S arrest as compared with the control and mock-transfected cells (Fig. 5).

**MOB1A overexpression induces cell proliferation, migration, invasiveness, and cell cycle of ovarian carcinoma cells.**

**Fig. 6:**
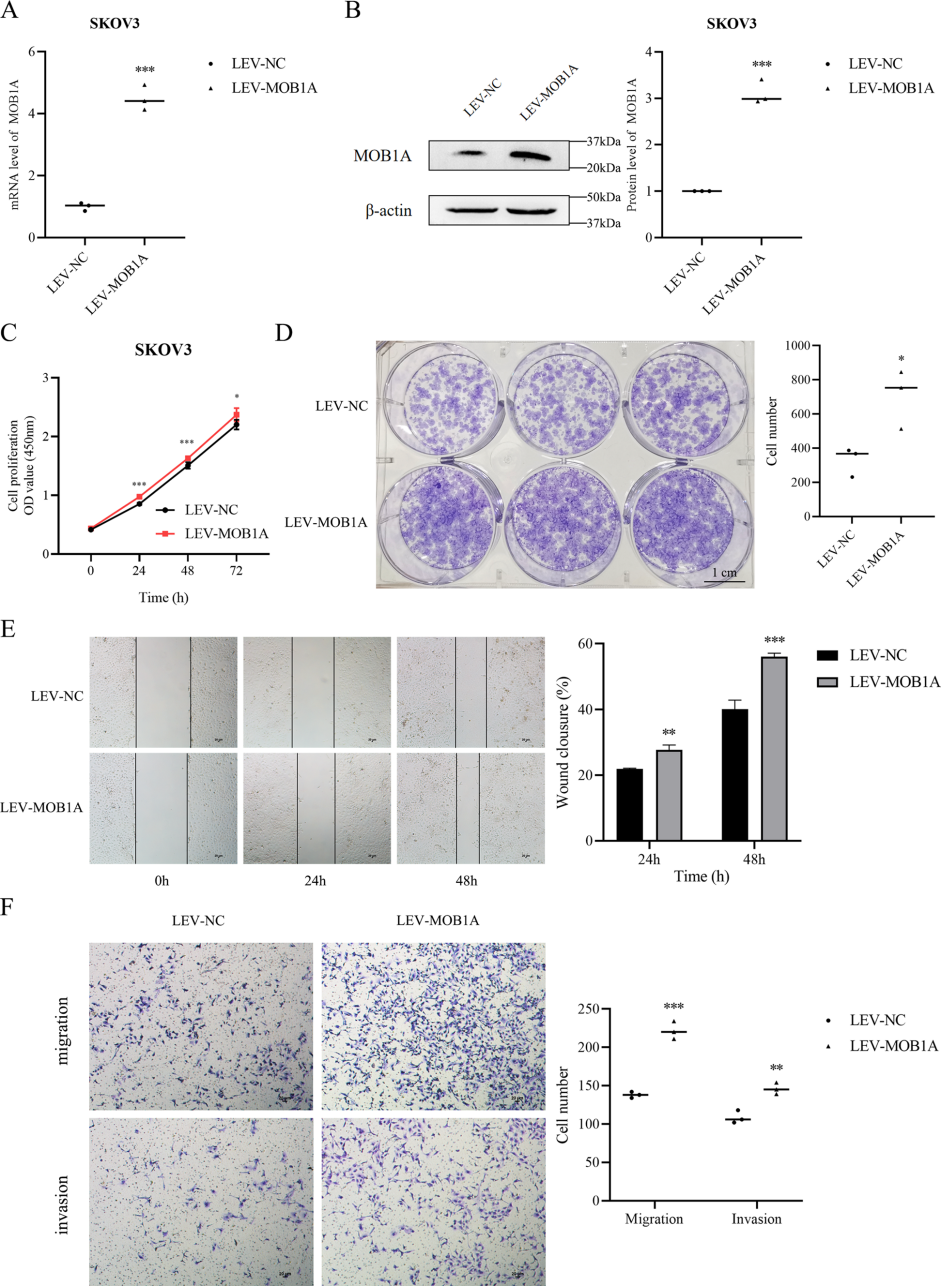
MOB1A overexpression induced ovarian carcinoma cell proliferation, migration, invasion ability, and cell cycle. Results showed the upregulation of MOB1A (A, B) induced the proliferation (C, D), migration, and invasion (E, F) of SKOV3 cells compared with the control groups (mean, N = 3). Bar scale: 1 cm (D), 20 μm (E and F). **P < 0.01, ***P < 0.001

Analysis by qRT-PCR and WB indicated that OC plasmid transfection increased MOB1A expression. The real-time cell analyzer (RTCA) assay showed that the viability of OC-transfected cells was increased as compared with mock-transfected cells (Fig. 6A and B). CCK-8 staining demonstrated significantly upregulated proliferation in cells overexpressing OC (Fig. 6C). We also detected the colony-forming of SKOV3 cells by plate clone formation assay. The results of plate cloning showed that the colony formation in the LEV-MOB1A group was increased (Fig. 6D).

Wound-healing assay was implemented to probe the corresponding function of MOB1A on the mobility of SKOV3 cells. We observed a significant difference in cell migration between LEV-NC and LEV-MOB1A cells. Cell migration in the overexpressing MOB1A group was notably superior to that in the LEV-NC group (Fig. 6E). Furthermore, a Transwell chamber assay was carried out to explore the connection of MOB1A with cell invasion in SKOV3 cells (Fig. 6F). The results showed that MOB1A was positively correlated with cell migration and invasiveness in SKOV3 cells.

**Fig. 7:**
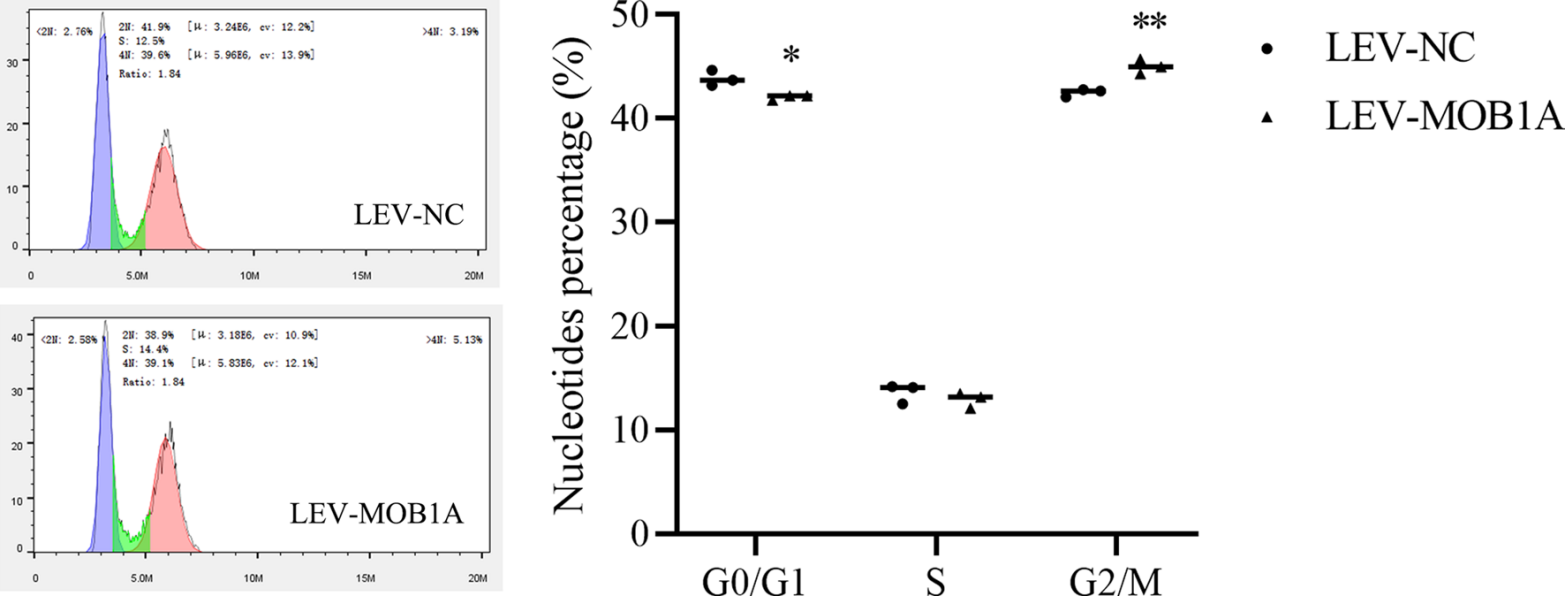
Up-regulated MOB1A expression promoted cell cycle progression in ovarian cancer (mean, N = 3). *P < 0.05, **P < 0.01

Cell cycle assays showed that MOB1A overexpression induced S/G2 progression as compared with the control and mock-transfected cells (Fig. 7).

### MOB1A reduces ovarian carcinoma cell tumorigenesis in vivo

**Fig. 8:**
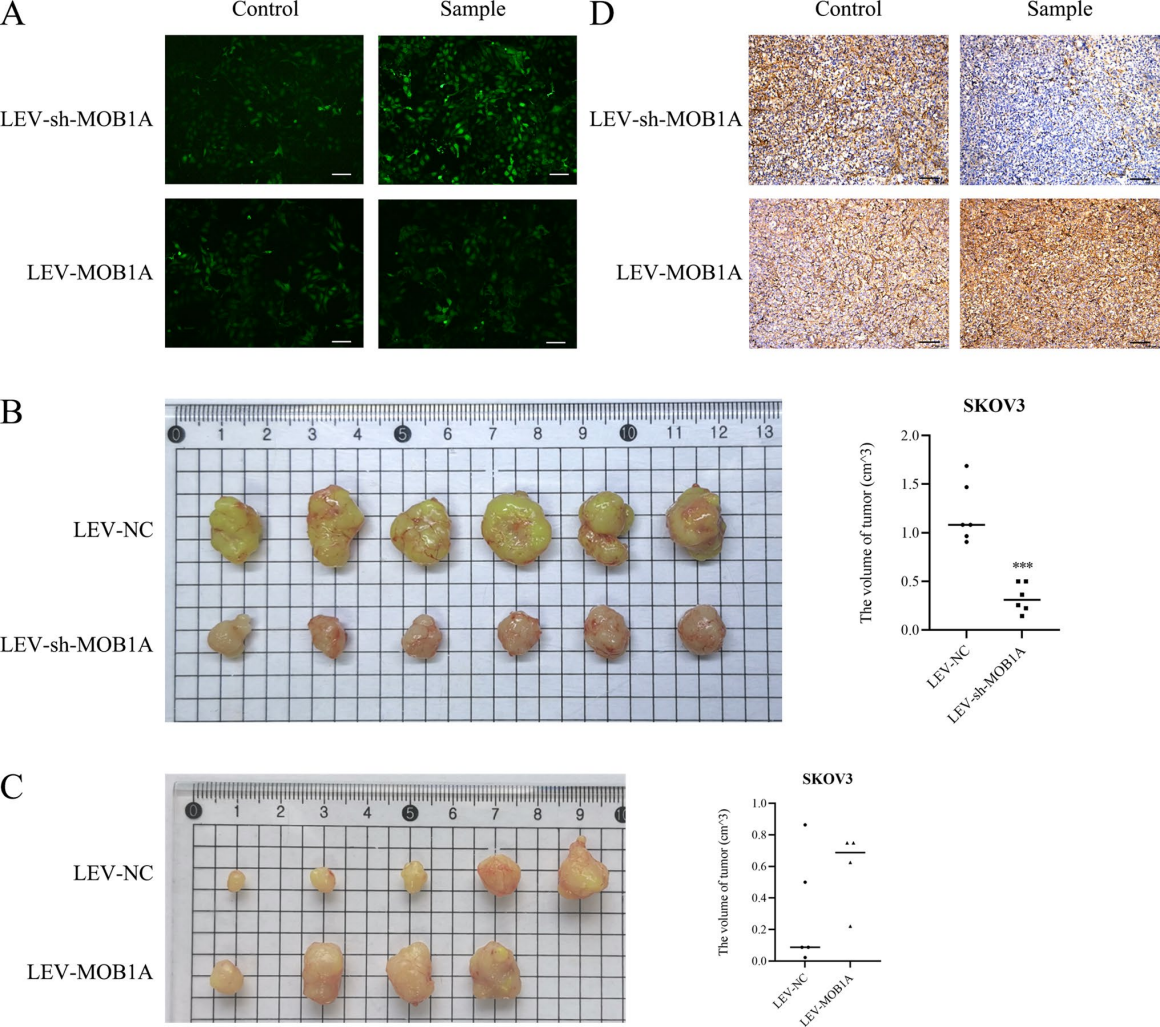
MOB1A downregulation and overexpression affected the tumorigenicity of ovarian tumor cells in vivo. **A** The efficiency of MOB1A silencing lentivirus and overexpression lentivirus transfection in SKOV3 cells; **B** SKOV3 cells with MOB1A downregulation reduced the volume of the tumor in vivo (mean, N = 6). **C** SKOV3 cells with MOB1A overexpression induced the volume of the tumor in vivo (mean, N = 6). **D** IHC analysis showed the expression of MOB1A in tumor tissues of downregulation and overexpression groups in vivo. Bar scale: 100 μm. ***P < 0.001

In this study, MOB1A down-regulated or up-regulated cell models were used for in vivo experiments (Fig. 8A). Female nude mice injected with MOB1A decreased cells showed a significantly lower tumor volume after inoculation compared with the control group (Fig. 8B). Immunohistochemistry (IHC) analysis showed significantly lower MOB1A expression in the MOB1A downregulated group compared with the control group (Fig. 8D).

**MOB1A overexpression induces ovarian carcinoma cell tumorigenesis in vivo**

In this study, female nude mice injected with LEV-MOB1A-SKOV3 overexpression cells showed a significantly larger tumor volume after inoculation compared with the control group (Fig. 8C). IHC analysis showed significantly higher MOB1A expression in the MOB1A overexpression group compared with the control group (Fig. 8D).

### Functional and pathway analysis of MOB1A

**Fig. 9:**
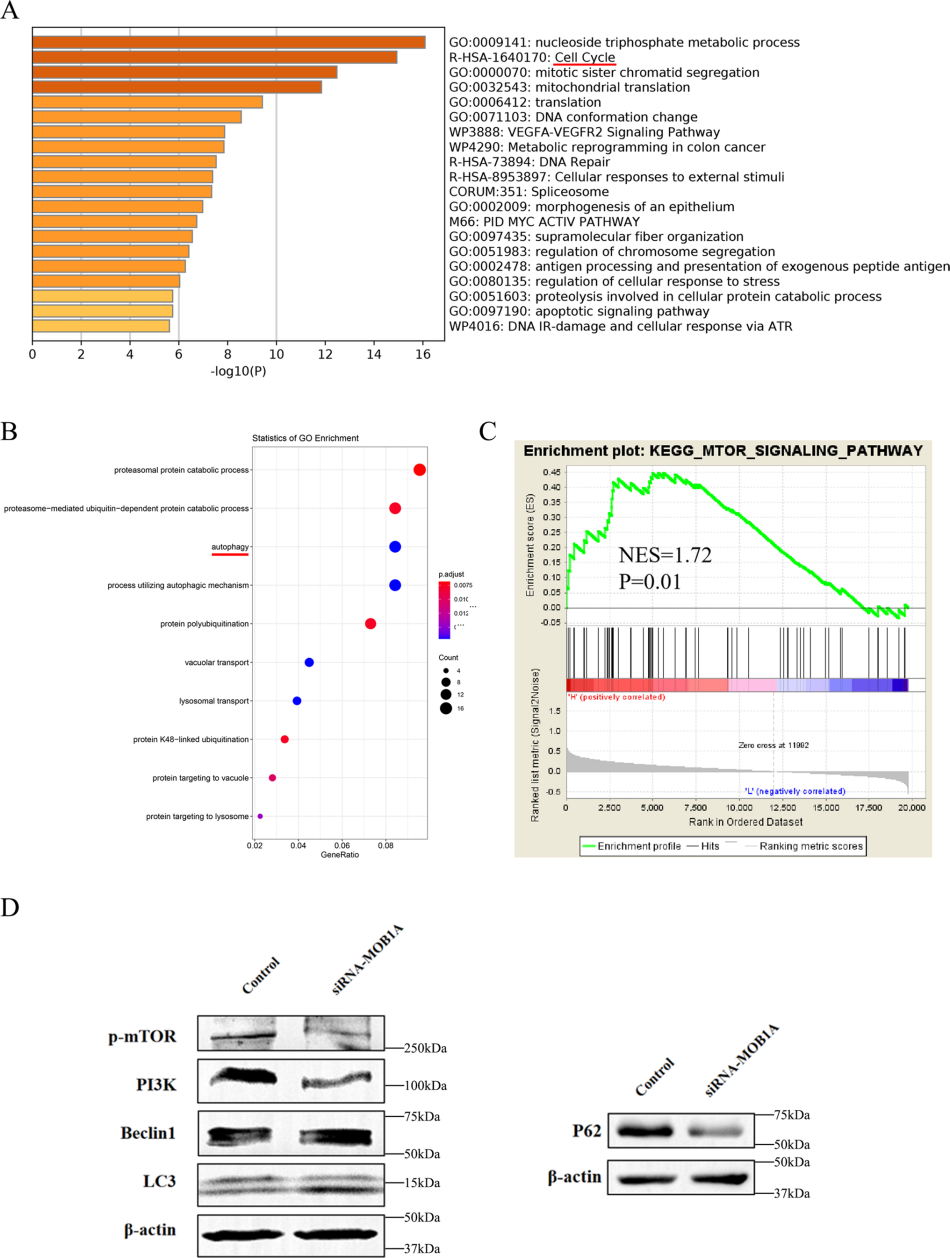
Functional and pathway analysis of MOB1A. **A** The functional analysis of MOB1A by Metascape; **B** The GO enrichment analysis of MOB1A by clusterProfiler package in R 3.6.3; **C** The gene set enrichment analysis of MOB1A by GSEA software 3.0; **D** Changes in the expression of p-mTOR, PI3K, Beclin1, LC3, and SQSTM1 (P62) protein in SKOV3 cells after transfection of MOB1A siRNA. The trials were repeated three times with the most significant results presented

For a deeper understanding of the role of MOB1A in OC, we obtained 200 genes positively associated with MOB1A expression in OC tissues from TCGA. Metascape and clusterProfiler packages were applied to run Gene Ontology (GO) and Kyoto Encyclopedia of Gene and Genome (KEGG) pathway enrichment analysis. Metascape showed these genes were mainly enriched in GO: 0009141 (nucleoside triphosphate metabolic process); R-HSA-1,640,170 (Cell Cycle); GO:0000070 (mitotic sister chromatid segregation); GO:0032543 (mitochondrial translation); GO:0006412 (translation); GO:0071103 (DNA conformation change); WP3888: VEGFA-VEGFR2 Signaling Pathway; WP4290: Metabolic reprogramming in colon cancer; R-HSA-73,894: DNA Repair; R-HSA-8,953,897: Cellular responses to external stimuli (Fig. 9A).

Moreover, GO enrichment analysis revealed that biological processes like GO: 0010498 (proteasomal protein catabolic process); GO: 0043161, (proteasome-mediated ubiquitin-dependent protein catabolic process); GO: 0006914, (autophagy); GO: 0061919 (process utilizing autophagic mechanism); GO: 0000209 (protein polyubiquitination); GO: 0007034 (vacuolar transport); GO: 0007041(lysosomal transport); GO: 0070936 (protein K48-linked ubiquitination); GO: 0006623 (protein targeting to vacuole); GO: 0006622 (protein targeting to lysosome) (Fig. 9B).

Further investigation of the biological signal pathways of MOB1A in OC was carried out in a single gene Gene Set Enrichment Analysis (GSEA). The results showed KEGG_MTOR_SIGNAL_PATHWAY was upregulated in the high MOB1A expression group (Normalized Enrichment Score [NES] = 1.72, P = 0.01) (Fig. 9C).

From the perspective of the tumor immune microenvironment, Cell-Type Identification by Estimating Relative Subsets of RNA Transcripts (CIBERSORT) algorithm was used to analyze the immune cell infiltration level of OC samples from different MOB1A expression groups. Significantly, the expression of MOB1A was positively correlated with the degree of infiltration of Neutrophils (P = 2.3e-04 ), NK_cells_resting (P = 0.03), and T_cells_CD4_memery_resting (P = 0.01), and negatively correlated with the degree of infiltration of B_cells_memory (P = 3.1e-3) and T_cells_follicular_helper (P = 0.03) (SuAdditional file 1: Fig. S1).

## MOB1A promotes ovarian cancer development by impacting on PI3K/AKT/mTOR pathway and autophagy

The mammalian target of rapamycin (mTOR) is a serine/threonine kinase with a central role as a downstream mediator in the PI3K/AKT signaling pathway [12, 13]. When the signaling pathway is activated, mTOR is phosphorylated at Ser2448 and it regulates several cellular functions such as proliferation, angiogenesis, differentiation as well as tumorigenesis [14]. In this study, we investigated the expression of related genes and proteins in PI3K/AKT/mTOR signal pathway and autophagy after MOB1A siRNA transfection. In MOB1A siRNA transfection of OC cells, p-mTOR, PI3K, and P62 protein expression was significantly lower than in the control, whereas expression of LC3-II and Beclin1 was significantly increased by MOB1A siRNA transfection compared with the negative control (Fig. 9D). Collectively, the silencing of MOB1A may reduce and inhibit PI3K/AKT/mTOR pathway and promote cell autophagy in OC.

## Discussion

Despite continued developments in the treatment of OC, only 19% of patients were diagnosed at an early stage due to the lack of specific clinical symptoms and rapid tumor progression [15]. Approximately 20–30% of OC patients would progress or develop disease recurrence in 6 months after completing chemotherapy and had a median OS of only 12–18 months [16]. Therefore, investigating the effective biomarkers and identifying their effects in the diagnosis, development, and treatment of OC is a matter of urgency.

In this study, we integrated the TCGA and CTPAC and obtained 303 up-regulated genes in OC. Subsequently, only eight genes (KRT7, ELF3, CRIP1, KRT19, SPOCK2, CKAP2, UNC5B, and MOB1A) were associated with poor OS from TCGA OC patients. The eight genes have been described to act as oncogenes in various human tumors and link to tumor progression [17–23]. However, the role of MOB1A in OC remains unclear, thereby we conducted further studies on the expression, biological function, and potential mechanism of MOB1A in OC.

As one of the core components of the Hippo signaling pathway, MOB kinase activator 1 A (MOB1A/MATS1/MOBKL1B) regulates organ development and size by regulating cell proliferation, differentiation, and apoptosis in various tissues and organs. It plays an important role in body development and organ balance [24, 25]. The role of MOB1A in human tumors is still controversial. For example, High expression of MOB1 in non-small cell lung tumors was related to disease recurrence, probably as a result of increased invasion by cancer cells [10]. MOB1A furthered the survival of gallbladder cancer by inducing autophagy to reduce cellular apoptosis and activate the IL6/STAT3 signaling pathway [11]. However, MATS1 (also known as MOB1A) expression is decreased in tumor tissue and its low expression is connected with tumor progress, invasion, and metastasis of colorectal cancer [7]. MOB1A was a low expression in pancreatic tumors and was an independent predictor of shorter survival [26]. In the present study, we found that the expression level of MOB1A was higher in OC and was associated with poor prognosis in OC patients. At the same time, after knocking down MOB1A of SKOV3 cells, its ability to proliferation, migration, and invasion is weakened, the cell cycle is blocked, and autophagy activity is enhanced, suggesting that MOB1A may serve as an oncogenic gene in OC. After further overexpression of MO1BA in SKOV3 cells, the malignant biological function of tumor cells was enhanced, meanwhile, the cell cycle and autophagy capacity showed a different trend from knockdown.

Autophagy is highly regulated in the catabolic process that involves lysosomal degradation of intracellular components, damaged organelles, misfolded proteins, and toxic aggregates, reducing oxidative stress and protecting cells from damage [27]. Autophagy plays a dual role in malignant tumors including OC, which is related to tumor type, microenvironment, treatment methods, and genetic factors [28]. OC cell autophagy-related signaling pathways are usually down-regulated and play a self-protective role in OC tissue. In this case, promoting autophagy can inhibit the progression of OC. Several studies have shown that autophagy can affect the biological behaviors of OC cells, including proliferation and apoptosis, as a tumor suppressor [29–32]. In this study, the overexpression of MOB1A inhibited the autophagy activity of SKOV3 cells, which was accompanied by the enhancement of tumor cell proliferation and the promotion of the cell cycle. Therefore, MOB1A may promote the malignant biological behavior of OC cells by influencing the activity of autophagy.

According to previous studies, the PI3K/AKT/mTOR signaling pathway is activated in 70% of OC patients [33]. PI3K/AKT/mTOR signaling pathway plays an important role in the proliferation, invasion, cell cycle, angiogenesis, and drug resistance of OC [34]. Therefore, inhibitors of PI3K/AKT/mTOR signaling pathways have become a new direction in the treatment of OC patients. In addition, PI3K/AKT/mTOR signaling pathway is also involved in autophagy [35]. Using the GO enrichment, we found that the expression of MOB1A was associated with autophagy. Moreover, the results of GSEA showed that KEGG_MTOR_SIGNAL_PATHWAY was upregulated in the high MOB1A expression group. According to the *vitro* study, we found that the PI3K/AKT/mTOR signaling pathway and cell autophagy were decreased in MOB1A knockdown OC cells, as well as the ability of cell proliferation, invasion, and migration. These results suggested that the MOB1A may affect autophagy activity through the regulation of the PI3K/AKT/mTOR signaling pathway, thus promoting the malignant behavior of tumor cells.

In general, we performed comprehensive bioinformatics analysis using a variety of public databases and found that MOB1A was highly expressed in OC and was associated with poor prognosis in OC patients. Subsequently, we took the lead in further studying the function and potential mechanism of MOB1A in OC cells. Interestingly, our results showed inhibition of MOB1A inhibited the proliferation, invasion, migration, and cell cycle of OC, and promoted cell autophagy, while upregulated MOB1A had the opposite effect in SKOV3 cells. Next, we also discovered the positive effects of MOB1A expression on tumor volume in animal tumorigenesis experiments. Finally, the results of functional enrichment analysis and WB assay indicated that the PI3K/AKT/mTOR signaling pathway may be the key signaling pathway for MOB1A acting as an oncogene in OC.

There are some limitations in this study. First, although we collected a large number of OC clinical samples from public databases, we still lacked our data support. Second, the gain-of-function experiment was not carried out in normal ovarian cells. Third, we did not use pathway inhibitors or activators to restore function. Finally, only the SKOV3 cell line was used in the present study, future studies using more OC cell lines are required to confirm the findings of our study. However, the current results of expression, prognosis, cellular biological experiments, and pathway mechanism after knockdown and upregulation of MOB1A have important value in clarifying the role of MOB1A in OC.

## Conclusions

This study demonstrates for the first time that MOB1A is highly expressed in OC and correlated with poor prognosis. MOB1A could regulate OC cell proliferation, invasion, migration, cell cycle, and autophagy through the PI3K/AKT/mTOR signaling pathway. These findings suggest that MOB1A may play an oncogenic role in OC and may serve as a potential therapeutic target.

### Future perspective

Overexpression of *MOB1A* has a positive effect on ovarian cancer progression and is likely involved in the regulation of the PI3K/AKT/mTOR signaling pathway. Our results pave the way for further study of the association between MOB1A and ovarian cancer. Future studies should be required to investigate clinical verification and molecular mechanisms. Clinically, more ovarian cancer samples should be collected to analyze how the expression of MOB1A is associated with the prognosis and development of ovarian cancer. The molecular mechanism exploration could pay attention to genetic alterations, protein interactions, and functional networks. Together, a detailed delineation of the molecular mechanism by which MOB1A modulates ovarian cancer development awaits future investigation.

### Summary points

- MOB1A is one of the members of the family of Mps One binder coactivator proteins.
- MOB1A is known to control organ size and tumor growth.
- The role of MOB1A in ovarian cancer remains unclear.
- MOB1A is significantly upregulated and is associated with inferior survival in ovarian cancer.
- M Inhibition of MOB1A inhibits the malignant process of ovarian cancer cells and induced cell autophagy.
- MOB1A upregulation had the opposite effects.
- MOB1A plays an important role in PI3K/AKT/mTOR signaling pathway.
- MMOB1A act as an oncogene by targeting PI3K/AKT/mTOR in OC.

## Materials and methods

### Cell lines and tissue culture

Three human OC cell lines, including Anglne, OVCAR3, and SKOV3, as well as the human normal ovarian epithelial cell line IOSE80 were used in this study. All the cell lines were acquired from Procell Life Science & Technology Company and were maintained in Dulbecco’s modified Eagle’s medium (DMEM; Shanghai Basal Media Technologies Co., Ltd.) supplemented with 10% fetal bovine serum (FBS; CellMax, Beijing, China), 100 mg/mL penicillin G and 50 µg/mL streptomycin (NCM Biotech, Zhejiang, China) within a humidified incubator containing 5% CO2 at 37 °C.

### Cell transfections and RNA interference

The SKOV3 cell line was selected for subsequent research and cells were maintained in 6-well plates with a density of 4 × 10^5^ cells per well. Cells were transiently transfected with MOB1A siRNA, si-NC (si-small interfering negative control) with a concentration of 60 nM by using Entranster-R4000 (Engreen Biosystem), following the manufacturer’s instructions. Assays were performed 48 h after transfection.

### Lentiviral vector production and generation of stable cell lines

The GFP-labeled lentivirus vectors containing the MOB1A silencing lentivirus, overexpression lentivirus, and the corresponding control lentivirus were obtained from Public Protein/Plasmid Library (Jiangsu, China). SKOV3 cells were transfected with various plasmids using lentivirus according to the manufacturer’s instructions. Stable clonal cell lines were selected with 3 µg/mL puromycin.

### RNA extraction and real-time polymerase chain reaction (RT-PCR)

Total RNA was extracted from cells using TRIzol reagent Aidlab Biotechnologies Co.,Ltd, Beijing, China) following the manufacturer’s protocol. cDNA was synthesized using the HiScript II Q RT SuperMix for the qPCR reagent kit (Vazyme Biotech Co.,Ltd, Nanjing, China) following the manufacturer’s protocol. RT-PCR was conducted by using the Hieff qPCR SYBR Green Master Mix (YEASEN, Shanghai, China), and the assay samples were processed using the ABI 7500 Thermocycler System (Applied Biosystems, CA, USA). GAPDH was supplied as a control, and each sample was verified in triplicate. The expression level was computed by the 2 − ΔΔCT method. The primers sequence was as follows:

GAPDH-F: 5′-CAGGAGGCATTGCTGATGAT-3′

GAPDH-R: 5′-GAAGGCTGGGGCTCATTT-3′

MOB1A-F: 5′- GCCGCTCTTCTAAAACATTCAA-3′

MOB1A-R: 5′- TCTCAGATTCCCACTTCCTAGA-3′.

### Western blotting

Cells were washed and collected and then directly lysed in RIPA buffer (Cwbiotech, Beijing, China) containing a protease inhibitor cocktail. Protein bands were separated by SDS-polyacrylamide gel electrophoresis and then transferred onto a polyvinylidene fluoride (PVDF) membrane. After blacking with 5% fat-free milk for 1.5 hat room temperature and then the membranes were incubated with the blot overnight at 4 °C. The membranes were incubated with secondary antibodies for 1 h at room temperature after washing three times. The protein bands were visualized with an enhanced chemiluminescence detection system (NCM Biotech, Zhejiang, China).

### Tumor xenograft models

Animal experiments were conducted after approval by the Institutional Animal Use and Care Committee of Xiamen University. Six-week-old female Nu/Nu nude mice were used in the *vivo* study and six nude mice were used in each group. SKOV3-LEV-sh-NC, SKOV3-LEV-sh-MOB1A, SKOV3-LEV-NC, or SKOV3-LEV-MOB1A cells (2 × 10^6) were subcutaneously injected into the right foreleg region. Tumor volumes were calculated as follows: length × width^2 × 0.5. The maximal tumor size/burden was not exceeded the permissible tolerance in the IACUC protocol (< 2 cm). Immunohistochemistry was performed to evaluate MOB1A expression. All the experiments were randomized.

### Cell cycle distribution

Cells were harvested and fixed with 70% ice-cold ethanol for 24 h at 4 °C. Subsequently, the cells were washed with cold phosphate-buffered saline (PBS) twice and then stained with propidium iodide (YEASEN, Shanghai, China). The cell cycle distribution was detected using flow cytometry.

### Wound-healing assay

Cells were cultured into 6-well plates until 90% confluence. After being aspirated using a 200 µL pipette tip and washed with PBS, then cultured in DMEM. A microscope was used to capture the images at 0, 24, and 48 h. The wound area was measured using Image J software (National Institutes of Health, Bethesda, MD, USA).

### Transwell invasion assay

Matrigel-coated transwell cell culture chambers (BD Biosciences) were used for the invasion assays. Filters were coated with 30 µl of basement membrane Matrigel at a dilution of 1: 8. Cells (5 × 10^4 in200 µL of serum-free medium) were layered in the upper chambers. The lower chambers contained 500 µl of complete medium serving as the chemoattractant. After incubation at 37 °C for 24 h, cells that invaded at the bottom of the upper chamber were stained with crystal violet and counted using an Olympus fluorescence microscope (Tokyo, Japan).

### CCK-8 assay and colony formation assay

Cell proliferation ability was analyzed by cholecystokinin octopeptide cell counting kit-8 (CCK-8, Beyotime Biotechnology, China) and colony formation assay. Cells were seeded and cultured into 96-well plates at a density of 200 µL or 1 × 10 ^ 4 cells per well in the CCK-8 assay. The plates were incubated at 37 °C for 2 h After 24, 48, and 72 h, 10 mL CCK-8 reagent was added to CCK8 assay each well. Optical density at 450 nm was read with a microplate reader 2 h later.

For the colony formation assay, 8 × 10^3 cells were seeded and cultured into a 6-well plate and suspended in DMEM containing 10% FBS. The medium was replaced every 3 days until 12 days to allow colony formation. The colonies were fixed with 100% methanol for 15 min and dyed with crystal violet for 10 min. The number of colonies was calculated from representative areas. All trials were achieved in triplicate.

### Immunohistochemistry staining

For immunohistochemical analysis, tumor tissues were fixed using 4% paraformaldehyde, dehydrated, embedded in paraffin, sectioned at 4 μm, and mounted on glass slides. The slides were deparaffinized, rehydrated, and incubated with 3% H_2_O_2_. A primary antibody was added, and the slides were incubated at 4 ℃ overnight. Subsequently, a secondary IgG antibody with an HRP label was added. Staining was performed using a 3, 3-diaminobenzidine (DAB) substrate kit for peroxidase reaction and counterstained with hematoxylin. The UltrasensitiveTM SP (Mouse/Rabbit) IHC kit (Mai-xin Biotechnology Co., Fuzhou, China) was applied to IHC detection, strictly following the manufacturer’s protocol. Finally, images were observed and captured with an optical microscope.

### OC patients’ sample source and clinical data

The transcriptional profiles of OC tissue and normal ovarian tissue were obtained from the GEO, GTEx, and TCGA datasets [36, 37]. Further, protein expression was obtained from CPTAC and HPA [38, 39]. The KM Plotter was applied to investigate the prognosis of MOB1A in OC [40]. CIBERSORT was used to analyze the relationship between MOB1A and immune cells [41]. Tumor IMmune Estimation Resource (TIMER) was applied to output the Cox regression results, based on clinical factors (age, gender, ethnicity, tumor stages) and gene expression [42].

### Functional and pathway analysis

The process and pathway enrichment analyses were carried out by using Metascape (Metascape, http://metascape.org) and clusterProfiler package by R 4.0.2 [43]. The gene set enrichment analysis of MOB1A was conducted by GSEA software version 3.0. P < 0.05 and | NES | > 1 were considered statistically significant [44, 45].

### Statistical analyses

The Spearman correlation coefficient was utilized to calculate expression correlations under SPSS 24.0. The Student’s t-test was performed using GraphPad Prism 8.0. *P* values < 0.05 were considered to suggest statistical significance. The survival curve was estimated by the Kaplan-Meier method. All experiments were repeated 3 times, and data were expressed as the mean from representative experiments.

## Supplementary Information

**Additional file 1: Figure S1. **The relationship between the level ofimmune cell infiltration and MOB1A expression in OC.

## Data Availability

The datasets used and/or analyzed during the current study are available from the corresponding author upon reasonable request.

## Abbreviations

MOB1A: MOB Kinase Activator 1
OC: Ovarian cancer
TCGA: The Cancer Genome Atlas
GTEx: The Genotype-Tissue Expression
CTPAC: Clinical Proteomic Tumor Analysis Consortium
DEGs: Differentially expressed genes
GEPIA: Gene Expression Profiling Interactive Analysis
KM: Kaplan–Meier
OS: Overall survival
PFS: Progression-free survival
PPS: Postprogression survival
WB: Western blot
HPA: The Human Protein Atlas
CCK-8: Cholecystokinin octopeptide cell counting kit
IHC: Immunohistochemistry
CIBERSORT: Cell-Type Identification by Estimating Relative Subsets of RNA Transcripts
GO: Gene Ontology
KEGG: Kyoto Encyclopedia of Gene and Genome
GSEA: Gene Set Enrichment Analysis
NES: Normalized Enrichment Score
DMEM: Dulbecco’s modified Eagle’s medium
FBS: Fetal bovine serum
PVDF: Polyvinylidene fluoride
PBS: Phosphate-buffered saline
TIMER: Tumor IMmune Estimation Resource
