# Supplementary material for: MOB kinase activator 1A acts as an oncogene by targeting PI3K/AKT/mTOR in ovarian cancer
Source: Discov Oncol. 2023 Jun 14;14:100. doi: 10.1007/s12672-023-00705-3 (PMC10267075; doi:10.1007/s12672-023-00705-3)

Figure 3E: MOB1A

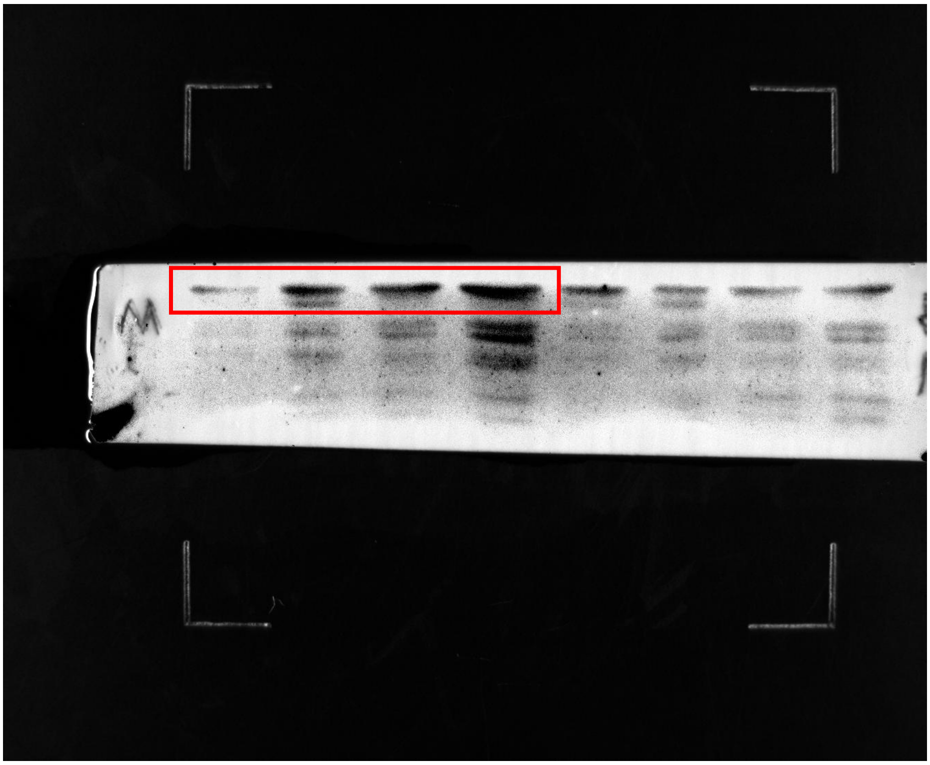

Figure 3E: β-actin

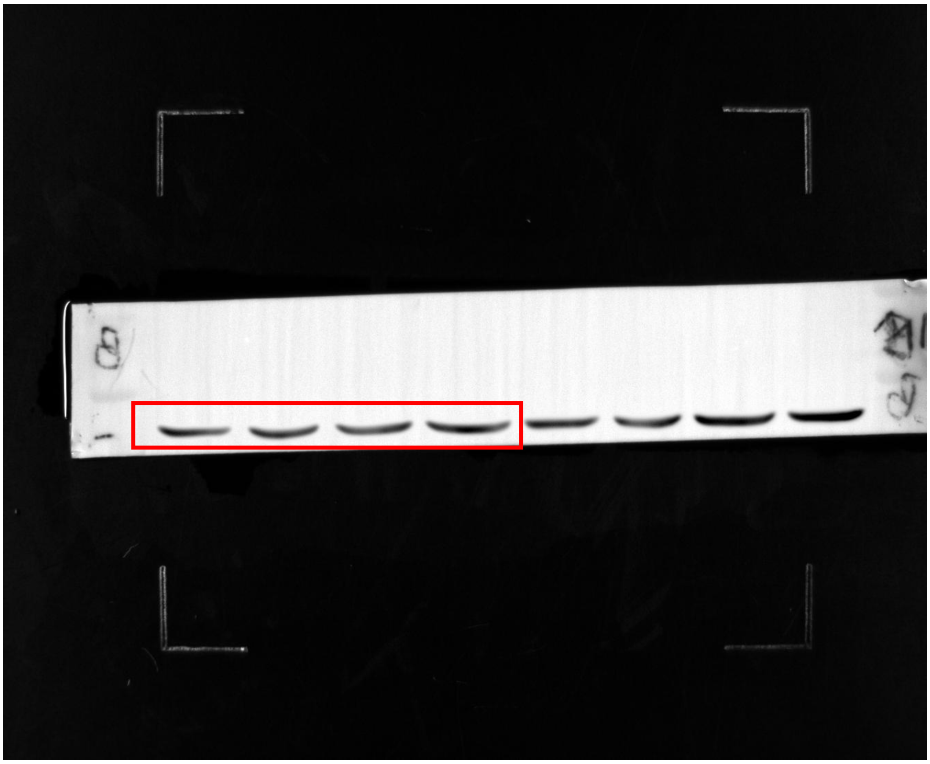

Figure 4B: MOB1A

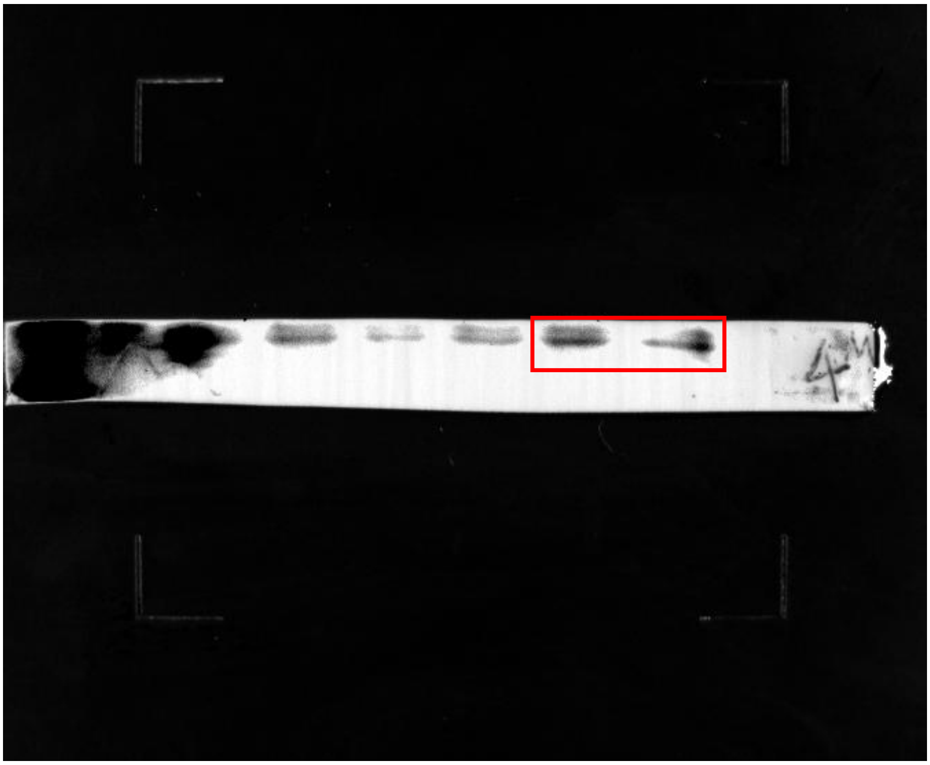

Figure 4B: β-actin and Figure 8: β-actin (right)

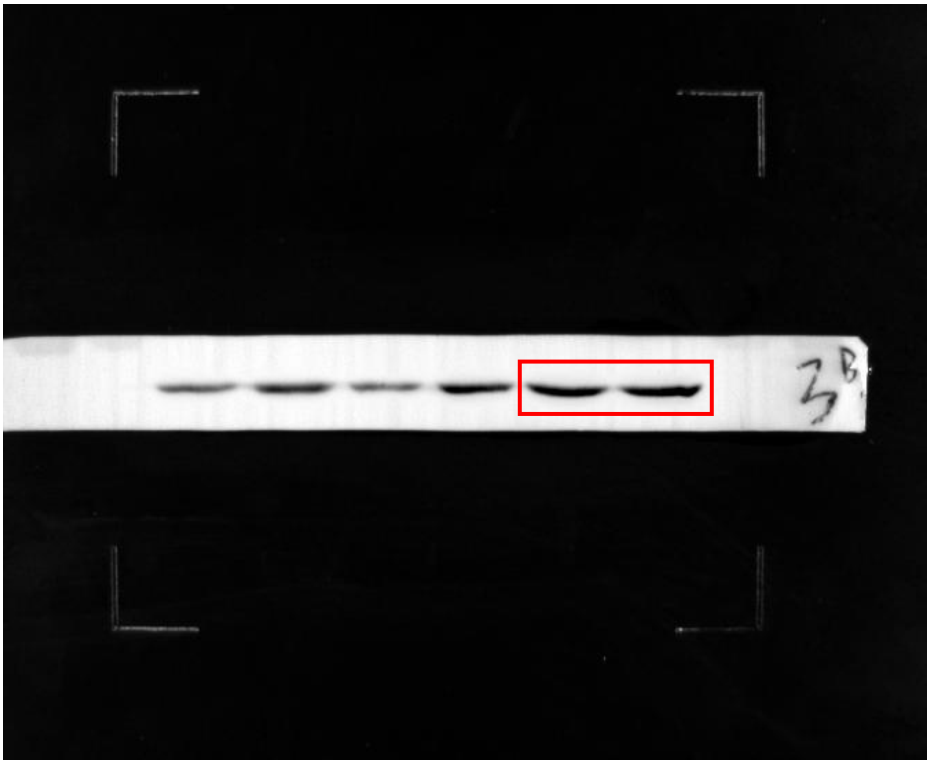

Figure 5B: MOB1A

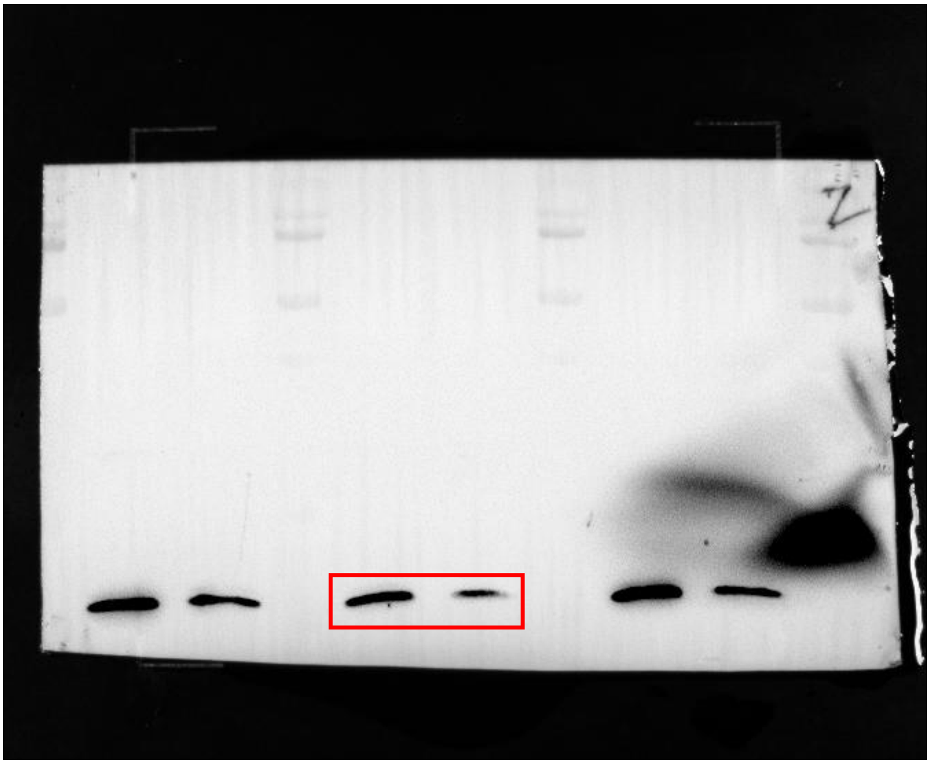

Figure 5B: β-actin

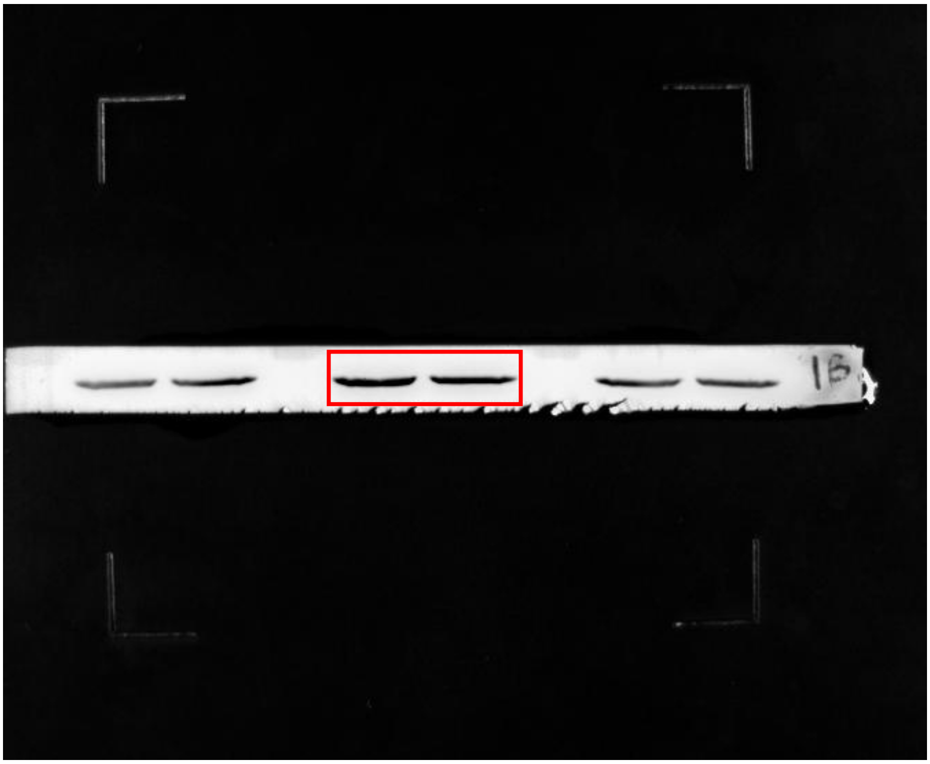

Figure 8: p-mTOR

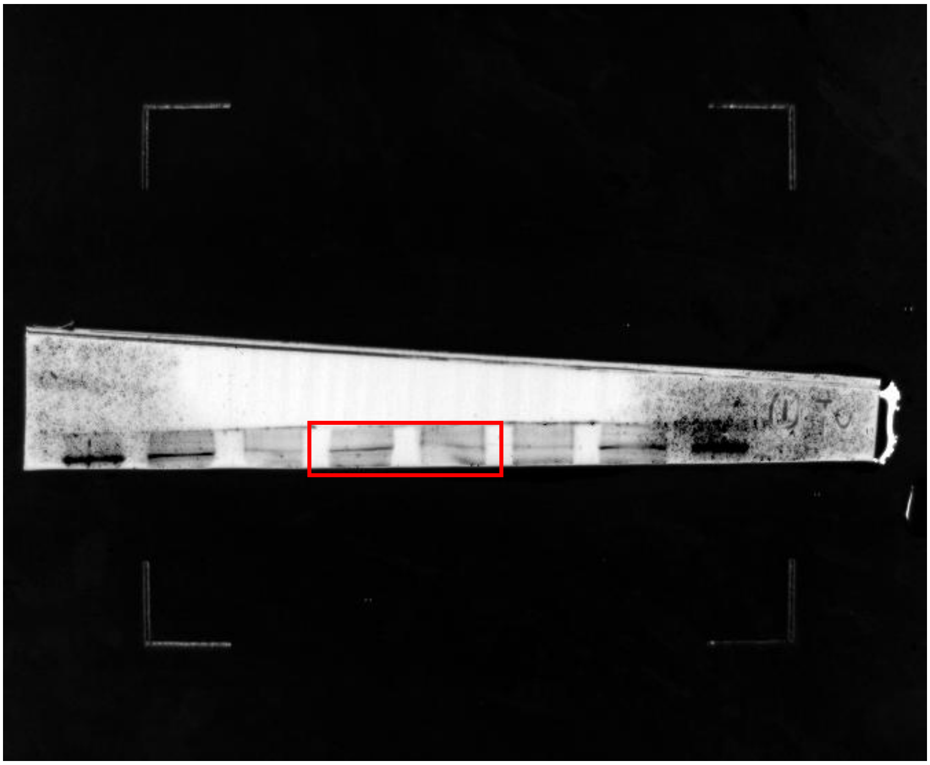

Figure 8: PI3K

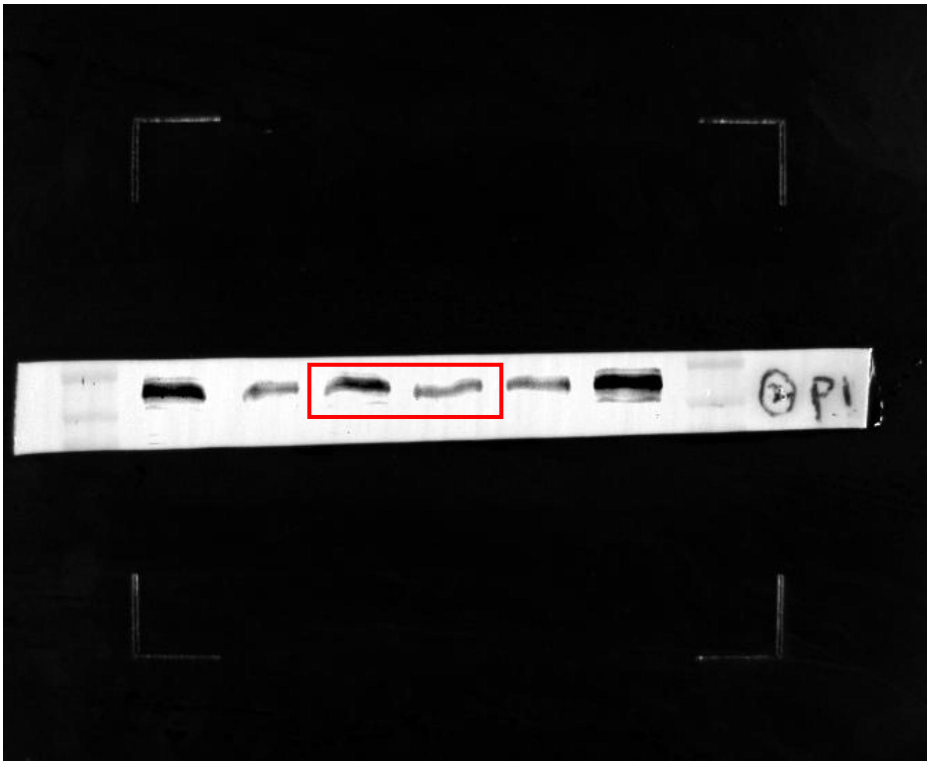

Figure 8: Beclin1

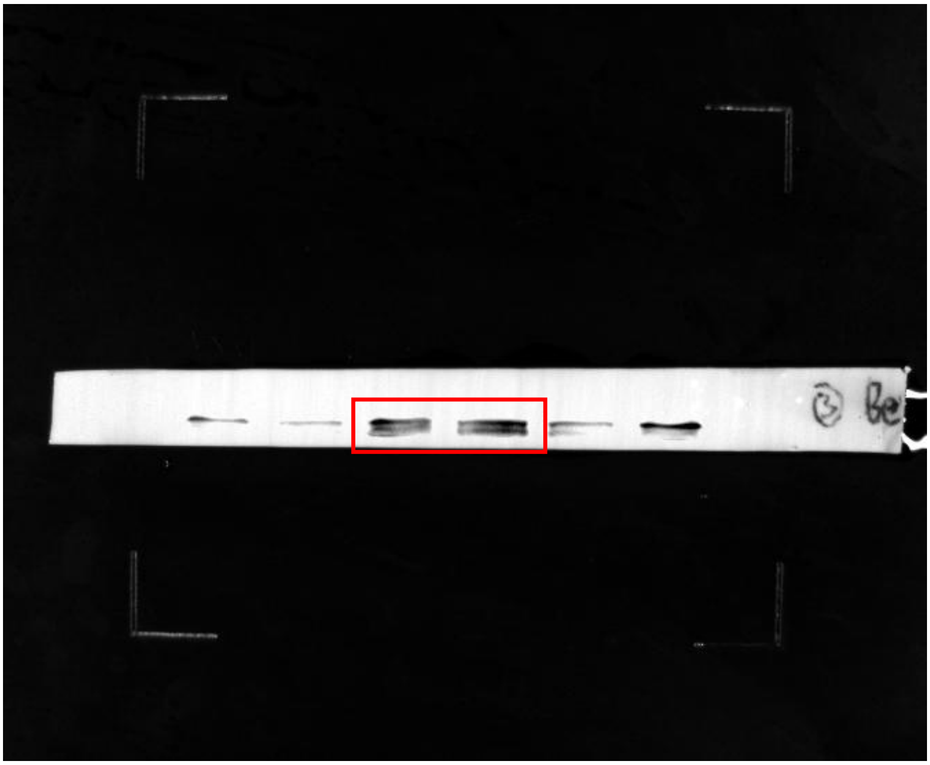

Figure 8: LC3

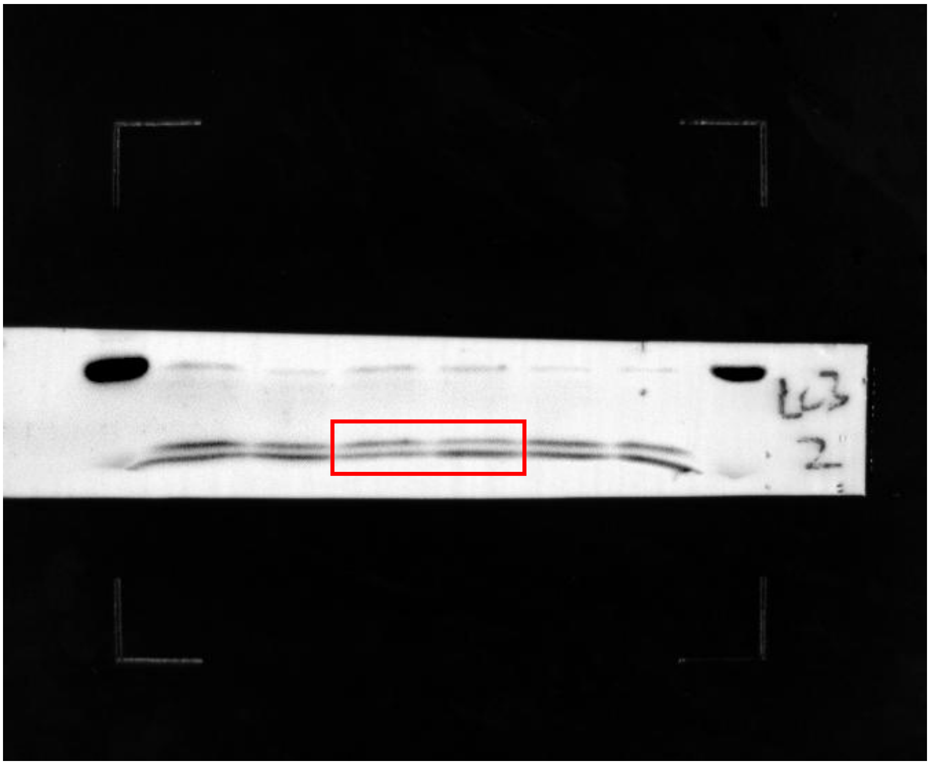

Figure 8: P62

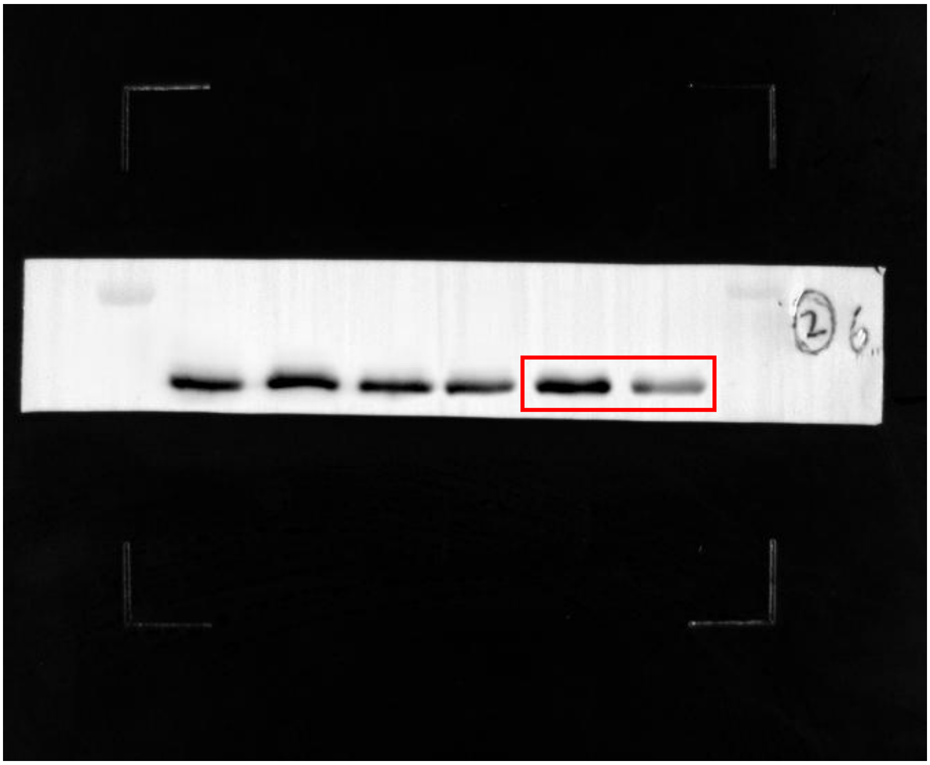

Supplementary Figure 1. The relationship between the level of immune cell infiltration and MOB1A expression in OC (CIBERSORT).

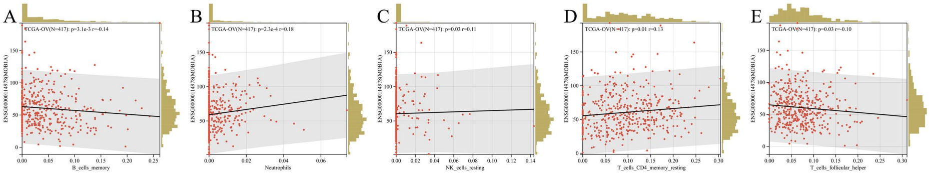

Supplement: Supplementary file 1 — Additional file 1: Figure S1. The relationship between the level ofimmune cell infiltration and MOB1A expression in OC. [file 12672_2023_705_MOESM1_ESM.pdf]
